# Round the Hospitals

**Published:** 1917-09-15

**Authors:** 


### ROUND THE HOSPITALS.

In this section, in our issue of September 1, we dealt with the questions underlying the policy of compelling four years' service from a nurse in a hospital, including her years of training, before granting her a full certificate. We gave a summary of what has taken place in Australasia, and the findings of Dr. Fetherston in his report prepared by the instructions of the Federal Government of Australasia, in which he stated that the addition of an extra year to the three years' period for training, in the case of a nurse, merely carries the system of sweating still further, his contention being that a three years' course in general hospitals should be ample time for nurses to acquire a thorough practical training. A valued correspondent who has had many years' experience in the training of nurses agrees that the question in this country has been treated heretofore largely as an economic question, from the authorities' point of view, in those hospitals which insist on four years' service before granting a full certificate of training. There is also the nurses' economic point of view to be considered. This is that in view of the general custom the four years' system imposes a heavy penalty on every nurse under it, and that it is economically unjust and unsound, so far as the nurses themselves are concerned. Our correspondent recognises these economic considerations and holds strongly that every training-school and every hospital connected with it should grant a three years' certificate at the end of the third year. The opinion held is that facilities should be offered to the better educated, more ambitious, and highly trained nurses to continue in the service of the hospital, and to perfect themselves in any special departments which they may affect, the hospitals employing them as sisters, or paying them in their fourth year at the higher rate which they give for fully trained nurses during the further period which these former probationers may devote to the completion of their training in the extra departments in question. We shall be glad to have correspondence on these aspects of nurse-training from any matrons, sisters, or trained nurses who may be interested in the points raised.

---

The College of Nursing promises to prove a material aid to the hastening of the millennium we have in prospect, and its arrival can be immeasurably quickened by every British trained nurse who gives her mind to the development of the College and enrols herself under its banner with the object of doing her part to help forward her profession, and so become entitled to share the good things ahead as they materialise. Our readers should keep constantly in mind the vital principle for which the College and the great body of nurses are contending. This is the direct representation of the nurses themselves on the College Council, that they may do their part to secure its triumphant success.

---

It would be interesting to know how far and to what extent Commandants and other responsible people in charge of V.A.D. hospitals and kindred

**ROUND THE HOSPITALS**—(*continued*).

establishments are under the impression that the most useful work they can do for the nation, at the present time, is to feed up and bring into good condition the soldier-patients sent to them. Where and how, exactly, this idea arose it would be interesting to know, and the fact that it exists at all supplies food for thought, which we hope may stimulate inquiry and secure results by the production of facts and actual experiences in this regard, which we should be glad to publish.

EDITING a newspaper during the war is no joke, for the position in war-time of an editor of a paper with a world-wide circulation is akin in the matter of time to that of many a matron and sister in these islands and farther afield. How many matrons are there in the position of one of the oldest and ablest in the country? From 7 A.M. until sometimes after midnight she is occupied. Her hospital contains over 120 patients, of whom fifty are soldiers, with only a Belgian house surgeon and a depleted staff to attend to them. The War Office multiplies forms to be filled up in duplicate by the matron, who has to do all this work in the special circumstances and has no assistant. (Will Sir Alfred Keogh please note, and, if possible, stop the multiplication of forms?) The five trained sisters under our friend are all fully occupied. Well may this matron say, "dreaming with myself is out of the question, and the days slip by."

HOW many other matrons are there in high position, who have laboured uncomplainingly and continuously, under equal pressure, since the war began? In the ordinary course many of them would help to lighten the Editor's work by co-operating and sending copy. How few of them can even contemplate, much less attempt to give this invaluable aid at present. Yet though matrons are too much taken up with getting through each day's work, given a subject or a point of special interest at the moment, or in the work which occupies the matron's mind, and she will find restful relief by committing her thoughts to paper and sending them to the Editor. So, knowing this to be the case, we shall hope, and continue to hope, for copy each week, for hope springs eternal in the Editor's breast. Each reader will sympathise with her fellow-worker, the Editor, and sympathy means remembrance, and remembrance often leads to the desire to join hands. So our letter-box is continuously occupied by interesting matter from an increasing number of matrons and sisters. Sometimes the nurse herself lends a hand, which is cordially welcomed.

AT a meeting of the Central Joint V.A.D. Committee held last week the following resolution was unanimously passed: "That a vote of thanks be, and is hereby, passed to Miss Swift, R.R.C., Matron-in-Chief of the Joint War Committee, for the valuable services rendered to the V.A.D. organisation by her numerous visits of inspection to auxiliary hospitals." We are pleased to record this official expression of a gratitude which Miss Swift has well deserved by her untiring and devoted labours in the cause of the efficiency of the numerous auxiliary hospitals up and down the country. The work has been hard and wearying, and has required great determination, energy, and tact in its execution.

THE *Times* records the death at a French Red Cross ambulance station in Flanders, of Sister Fanny Kessissoglu, who had worked indefatigably with the French unit of the Secours aux Blessés all through the Balkan campaign. She rejoined her unit under Mme. Panas and worked with great bravery on the French Front, being awarded the Croix de Guerre. On two occasions she was mentioned in despatches. Her death was contributed to by the nature of her work, which weakened her constitution. Sister Kessissoglu was buried with military honours at Zuydecoote.

MANIFOLD are the details that go to the adequate and efficient equipment of our soldiers at the various Fronts. A minor detail, but one that contributes no little to the soldier's comfort and contentment, is the hospital bag in which he keeps his treasures and small possessions, which thus remain always with him. Lady Smith-Dorrien, who has organised the manufacture and supply of these bags at the request of Sir Alfred Keogh, has to meet a normal demand for 120,000 per month, which may be increased from time to time, as was the case last month, when 10,000 extra bags were asked for by the Director of Medical Services in France. The work of receiving and forwarding these bags is carried on at 26 Pont Street, London, S.W. 1, in a house lent to Lady Smith-Dorrien's Hospital Bag Fund by Mr. Cox, of Cox's Bank. The bags are for the most part made by workers outside, who by sending 7s. 4d. to Lady Smith-Dorrien receive in exchange sufficient cretonne, tape, and labels for thirty bags, carriage free, together with a sample bag to show the correct way of making. The bags are simple to make, easy to handle, and cheering to look at in the process, being of gaily coloured and patterned cretonne. Lady Smith-Dorrien has a large band of voluntary workers, who are not able to pay for materials but are glad to give their time to the work, and for this purpose donations of any amount are thankfully received. We have no doubt there are many of our readers who will be glad to help Lady Smith-Dorrien in this undertaking, either by sending a donation or offering to make a certain number of the hospital bags, or even doing both. They should in either case write to her at 26 Pont Street, London, S.W. 1. (Telephone: Kensington 1072), when the fullest information will be sent them. The aim is to secure a large number of people, each undertaking to send in regularly, at intervals of, say, a fortnight or a month, a certain number of bags. At present Lady Smith-Dorrien has a stock of cretonne bought at 7d. a yard, but prices are rising daily, and when the present stock is exhausted higher prices will undoubtedly prevail.

For "The British Nursing Profession: The Two Voices," see p. 497; "Nursing Affairs in Ireland," p. 492; Matrons' and Sisters' Appointments, p. 498.

